# PROTOCOL: Is radicalization a family issue? A systematic review of family‐related risk and protective factors, consequences, and interventions against radicalization

**DOI:** 10.1002/cl2.1190

**Published:** 2021-07-26

**Authors:** Izabela Zych, Elena Nasaescu

**Affiliations:** ^1^ Faculty of Educational Sciences and Psychology University of Cordoba Cordoba Spain

## Abstract

This systematic review aims to answer the following research questions: (1) What are the family‐related risk and protective factors for radicalization? (2) What is the impact of radicalization on families? (3) To what extent are family‐based interventions against radicalization effective? The review will answer these research questions by systematically gathering and synthesizing published and unpublished scientific literature on family‐related risk and protective factors for radicalization, the impact of radicalization on family, and studies that evaluate the impact of family‐based interventions on radicalization. This review will also explore what components of family‐based interventions are most effective for countering radicalization. Thus, this systematic review will provide a global vision of scientific literature focused on family and radicalization including quantitative research.

## 1 BACKGROUND

### 1.1 The problem, condition, or issue

Radicalization to violence is a complex sociopsychological process through which people acquire a series of extreme beliefs, attitudes, and ideologies, justifying the use of violence to achieve their goals and promote their ideologies (Borum, 2012; Doosje et al., 2016). Radicalization to violence is extremely harmful to social groups and the society as a whole, and it was found to be related to terrorism (Dugas & Kruglanski, 2014). Terrorism is one of the most important threats faced by the 21st century societies. Thus, countering radicalization to violence has become one of the most important national and international policy priorities and a crucial public safety issue worldwide.

Several studies (e.g., Neumann, 2013; Schmid, 2013) suggest that radical thinking and attitudes do not necessarily imply violent behavior. Purely cognitive radicalization is not problematic per se, and radical beliefs are a part of any healthy democratic society, becoming a problem if they are expressed through violent actions (Neumann, 2013). Radical violent behaviors are usually displayed only by a small number of radicalized individuals. Based on the two‐pyramids model (McCauley & Moskalenko, 2017), radicalization of opinion should be distinguished from radicalization of behavior. Although radical beliefs are not necessary or sufficient for becoming a terrorist (Schuurman & Taylor, 2018), it is usually assumed that individuals who engage in terrorism and radical violence show radical thinking first. For example, the staircase theory states that the process leading to terrorism is similar to a narrowing staircase where radical ideas appear before terrorist acts that occur “at the top of a building” (Moghaddam, 2005, p. 161). Thus, radical thinking can escalate to radical violence employed to achieve ideological, political, religious, social, or economic goals. This becomes a security threat because radical violent behaviors are justified by some individuals and groups as a way to promote extremist attitudes and ideologies (Doosje et al., 2016). It is therefore important to reduce both radical thinking and also radical behavior. In this systematic review, we use the term radicalization to refer to a cognitive or behavioral process resulting in either radicalism, extremism, or terrorism.

Some of the efforts to describe and understand radicalization focus on family as “potentially being risky, as well as potentially being a source of protection and rehabilitation” (Spalek, 2016, p. 46). The role of the family often differs considerably from case to case. While some families might provide protective factors by their resources, positive parenting or developing resilience toward radicalization (Radicalisation Awareness Network [RAN], 2017; Spalek, 2016), other families might provide risk factors by their poor resources and relationships or a direct undesirable ideological influence (King et al., 2011; Speckhard & Akhmedova, 2005). Family might not only facilitate and support radical and violent extremism activities (King et al., 2011), but more importantly, it might have a key role in preventing young people from radicalization and recruitment to violent extremist groups (RAN, 2017). Thus, families play an important role in radicalization, but empirical findings on the topic are inconclusive and a comprehensive research synthesis could clarify the role of family factors in radicalization.

The purpose of this systematic review is to provide a comprehensive research synthesis of existing empirical studies on family‐related risk and protective factors for radicalization, the impact of radicalization on family, and family‐related interventions against radicalization to build evidence‐based knowledge and guide future research, policy, and practice. A comprehensive synthesis of family‐based intervention programs will make it possible to discover what is already being done and what works best. Discovering and understanding family‐related risk and protective factors for radicalization, and consequences of radicalization for family, will advance knowledge of the etiology and impact of radicalization which will contribute to the improvement of prevention and intervention programs. Thus, this systematic review has three complimentary objectives of reviewing both risk and protective factors, consequences, and interventions. A research synthesis can provide a global panorama of the field that cannot be obtained through singular empirical studies given the limited number of participants and variables that can be included in each project.

### 1.2 Family‐focused risk and protective factors for radicalization to violence

This systematic review will include studies focused on family‐related risk and protective factors for radicalization. Both published and unpublished studies will be included if they provide enough information to calculate the effect size of the relation between radicalization and each family‐related variable conceptualized as a risk factor or a protective factor. Strictly speaking, a risk or a protective factor refers to a variable that associates with and precedes an outcome that should be compared between the affected population and general population free of the outcome of interest (Kraemer et al., 1997). Nevertheless, we anticipate that many of the included studies are cross‐sectional and therefore measure theoretically defined risk and protective factors.

In this systematic review, family‐related risk factors for radicalization are defined as variables related to childrearing, family structure, family violence and radicalization, and similar family‐related variables, that increase the risk of radicalization of opinion and behavior (e.g, corporal punishment by a parent, bullying by siblings). To be considered a risk factor, the associations should be tested by comparing radicalized individuals to a nonradicalized group.

Protective factors refer to variables that relate to low probability of negative outcomes (Lösel & Farrington, 2012). Family‐related protective factors are defined as variables related to childrearing, family structure, family violence, and radicalization, and similar family‐related variables, that decrease the risk of radicalization of opinion and behavior. Some specific examples could include parenting practices, parental warmth and involvement, marital status, among other family‐related variables.

Family factors can be crucial for radicalization based on several theories and research findings. Among them, Sageman (2004) found that social networks, including families, were important in explaining terrorist actions, attributing this fact to group influence on individual actions that is a well‐known phenomenon in social psychology. Moreover, parents guide behaviors of their children and explain the standards of behaviors considered appropriate (Bandura, 1991). A study by Zych et al. (2020) showed that parental induction of moral disengagement, where children are told that immoral actions can be justified, was related to violent behaviors in children. Thus, some parenting practices and expression of radical ideas by parents could induce their children to adopt radical attitudes and behaviors. On the other hand, other parenting practices, or expressions of ideas against radicalization could be protective.

Intergenerational transmission of antisocial behaviors was confirmed in several studies (Farrington et al., 2009). This is usually explained by social learning theories according to which children treat parents as models and imitate their behaviors. It can also be true for the relations with other family members. A qualitative study based on interviews with violent extremists showed that children raised in extremist families are at higher risk of becoming violent extremists themselves (Schils & Verhage, 2017). Moreover, some structural factors such as unemployment relate to radicalization (Siedler, 2006) as these issues can potentially make it harder for families to be informal social control handlers. Although the structural factors are beyond the scope of this systematic review, many of them are important to explain why families should be studied in relation to radicalization.

Family‐related risk and protective factors could also be related to radicalization in an indirect way. During child development, parents have a crucial role in promoting emotional health and well‐being, including a positive sense of self, skills to cope with stressful situations, regulate emotions, control fears, or accept frustrations. Also, parents' ability to encourage children's sense of belonging is crucial for their early development which could decrease radicalization. Several studies indicated that, among other factors, low sense of belonging makes young people more vulnerable to engage in violent and nonviolent radicalization (Borum, 2004; Ventriglio & Bhugra, 2019). Moreover, research suggests that low parental support, supervision, inconsistent parenting, or contact with family members with radical views enhance young people's vulnerability to radicalization (Sikkens, Sieckelinck, et al., 2017; Van Bergen et al., 2016). Parenting is therefore related to mental health, individual and social well‐being which can become risk or protective factors for radicalization.

Although family‐focused risk and protective factors were found among the strongest and most robust predictors of different antisocial behaviors including delinquency (Zych et al., 2021), there are still pressing gaps in knowledge related to family and radicalization. The existing studies suggest that there are different family‐related variables, including risk and protective factors, that relate to radicalization (Harris‐Hogan, 2014; Schmid, 2013). Among them, a study conducted with Swiss adolescents (Nivette et al., 2017) focused on the relation between parental involvement in adolescent's everyday life and violent extremist attitudes. It is possible that parental involvement contributes to positive youth development at home and in communities, which could prevent radicalization. Siedler (2006) studied if parental unemployment during childhood and parental leaning toward right‐wing extremism were related to right‐wing extremism in their children in Germany. In the United States, Jasko et al. (2016) studied if having a family member involved in radical activities was related to the commitment of ideologically motivated crimes and if this relation existed after controlling for covariates such as age, gender, race/ethnic minority status, immigrant status, education level, military experience, and previous criminal activity. A study conducted in Iraq (Dhumad et al., 2020) focused on the relation between being convicted for terrorism and family factors such as an authoritarian father, disintegrated family, or having a family member that had been murdered.

Sikkens, Van San, et al. (2017) also explored the influence of family and the socialization context on radicalization. Based on interviews with both former extremists and their family members, no direct influence of family on radicalization was reported, although it was suggested that parents may influence the radicalization process indirectly. Moreover, Sikkens, Sieckelinck, et al. (2017) conducted a study aimed at examining parents' reaction when children developed radical ideology and concluded that parents often lack skills and strategies to cope with the situation. It is therefore possible that parental lack of reaction and response could facilitate children's radicalization. Thus, there are some empirical studies that identify family factors related to radicalization, but they provide inconsistent evidence regarding risk and protective factors.

Most of these studies provide evidence focused on specific risk and protective factors in specific contexts, and a research synthesis is needed to establish what is known and unknown. It is expected that undesirable family relationships (e.g., conflict), parenting practice (e.g., corporal punishment), and influence (e.g., radicalization of other family members) are related to more radicalization.

### 1.3 Impact of violent radicalization on families

The family has a crucial role as a socialization context that can provide emotional support and influence social identities of its members. Nevertheless, family can also have an undesired influence on its members (Zych et al., 2020). Radicalization of a family member could negatively impact other family members, but empirical studies focused on the impact of radicalization on families are inconclusive.

In this systematic review, family‐related consequences are defined as variables related to the psychological, physical, and structural impact of radicalization on families (e.g., divorce, mental health issues of family members). Once again, it would be ideal to base this part of the systematic review on longitudinal studies where radicalization precedes consequences. Nevertheless, many studies in the field are cross‐sectional and therefore, they will be included if they focus on family‐related consequences on a theoretical basis.

Radicalization might have damaging psychological and social effects on the family (Guru, 2012). Some studies suggest that families of radicalized individuals are victimized by others as they may become socially isolated (Gielen, 2015; Guru, 2012). Regarding consequences of radicalization for families, research shows that family members of radicalized individuals are frequently shamed, blamed and socially rejected which can be related to mental health issues (Guru, 2012). Labeling is a well‐known phenomenon in social sciences, according to which individuals start to behave according to labels given to them by others (Scheff. 1974). Labeling was found to be related to intergenerational transmission of crime (Besemer et al., 2017), and it is possible that family members of radicalized individuals are labeled. Labeling could be one of the mechanisms through which radicalization impact family members.

Family members of radicalized individuals can suffer internalizing problems such as anxiety and depression (Guru, 2012). This might lead to a polyvictimization process that could increase the risk of radicalization of the previously nonradicalized family members over the lifespan. Moreover, secondary victimization occurs when a victim suffers additional harm, being treated in an unfair way, including victim‐blaming attitudes (Williams, 1984). It is possible that family members of radicalized individuals suffer an indirect harm through secondary victimization.

Having a radicalized family member can be an overwhelming experience. Moreover, radicalized individuals focus on specific goals and sometimes “family and relationships are forgotten” (Kruglanski et al., 2014, p. 71). According to Sampson and Laub (1995), families are important resources to draw on during life transitions and turning points. Thus, if a family member is focused on radical goals, ignoring other aspects of life including the family, these important resources can be lost. Moreover, radicalization of a family member can have negative consequences as families can draw on resources based on extremist ideas that can negatively affect social and personal wellbeing. Social capital has been defined by Coleman (1988) as social structures that facilitate certain actions within the structures, making it possible to achieve certain goals. Social capital is based on trust and there are certain norms within social structures. If a family member becomes radicalized, the whole structure is likely to be affected. Some prosocial goals may become frustrated and other antisocial goals may appear. Thus, negative consequences of radicalization for family members are likely.

### 1.4 Family‐focused interventions for countering radicalization

#### 1.4.1 The intervention

There are some family‐based interventions conducted to decrease radicalization. For example, an 18‐months long pilot project “Ending Terrorism Through Youth Service Action Locally” (ETTYSAL) in Tunisia funded by the U.S. State Department and implemented by Creative Associates International (n.d.) focused on the importance of the family as a protective factor against radicalization. In this program, 100 Tunisian young people were evaluated for vulnerability to join extremist groups based on 12 risk factors (antisocial tendencies, poor parental supervision, family radicalization, critical life events, impulsive risk‐taking, neutralization of guilt, deviant behaviors, peer influence, peer radicalization, religious extremism, and social vulnerability). The intervention was individualized and focused on family counseling and group activities. ETTYSAL was evaluated to discover if this family‐centered intervention approach reduced the risk factors for radicalization to violent extremism after the program and check if decreasing family radicalization was an important risk factor that contributed to this decrease.

Effectiveness of family counseling was studied also in programs carried out in Europe. In Germany, *Hayat* (means “life” in Turkish) is a prominent program based on family counseling (Koehler, 2013). It aims at reducing violent and nonviolent radicalization at any stage. Despite some encouraging results, the effectiveness and impact of this intervention program against radicalization still needs to be assessed.

According to Radicalization Awareness Network, evaluation of the effectiveness and impact of the intervention programs is one of the pressing gaps in the literature regarding radicalization (Pisoiu & Ahmed, 2016). Thus, it is imperative to evaluate the existing family‐related intervention programs against radicalization through a rigorous methodology (Feddes & Gallucci, 2015). There is still much to be addressed and learned about how to better design, implement, and evaluate effective intervention programs against radicalization. A systematic review will help better understand the state‐of‐the‐art and identify which family‐related components of intervention programs showed evidence to be effective against radicalization.

In this review, family‐based interventions will refer to any activity, strategy, technique, training, and program that involves family as a recipient of the intervention or to the interventions related, focused, or targeted on family‐related risk and protective factors to decrease radicalization. The included studies will be based on randomized controlled trials and quasi‐experiments. It is expected to discover which components of family‐based interventions are effective against radical attitudes and behaviors. Any intervention program that focuses on family is eligible (e.g., siblings, parents, extended family) if cognitive or behavioral radicalization, extremism or terrorism are measured as the outcome variable. Intervention groups will usually be compared to control groups (with no intervention).

It is important to conduct a systematic review of family‐focused interventions to understand what is known and what needs to be discovered next. A research synthesis will provide a global panorama and an up‐to‐date evidence about family‐based interventions against radicalization and, if a systematic review concludes that the number of high‐quality studies is low, this could encourage new primary research with high‐quality methodology.

#### 1.4.2 How the intervention might work

Family‐based interventions can be effective if they focus on family related risk and protective factors. If family is one of the most meaningful groups for individuals, and groups influence individual's behaviors including terrorist acts (Sageman, 2004), family‐based interventions could promote desirable goals and deradicalization. Given that parents guide children's behaviors (Bandura, 1991), it is possible that family‐based interventions promote desirable parental influence. Interventions can also improve social capital based on families and provide resources against radicalization (Koehler, 2015). Moreover, interventions could improve family's capacity to promote self‐control, which is especially important because low self‐control is related to antisocial behavior, and its level is influenced by family (Gottfredson & Hirschi, 2020). According to social control theory (Hirschi, 1969), antisocial behavior is inhibited by strong and long‐term bonds with others, including parents. Thus, family could be key to understanding and preventing radicalization, but more research is needed to confirm this.

In this systematic review, we synthesize knowledge regarding family‐based interventions to prevent radicalization, and also interventions focused on deradicalization. Interventions could focus on increasing protective factors and decreasing risk. Studies focused on interventions are different from studies on protective factors because they include an explicit manipulation of independent variables by the intervention providers.

### 1.5 Why it is important to do this review

Despite a growing body of research on radicalization, studies focused on family‐factors are still in their early stages. A family‐specific focus, including parents, siblings, children, spouses, and extended family members could fill pressing gaps in knowledge that would make it possible to understand family impact on radicalization, including its cognitive and behavioral components. In our systematic review, family is defined as a group related by consanguinity, adoption, marriage, and similar long‐term couple relationships.

There are several research syntheses focused on radicalization, but none of them focused specifically on family‐related variables and interventions. Among them, a systematic review focused on protective factors against extremism and violent radicalization was published by Lösel et al. (2018). This systematic review was based on comprehensive searches in 15 databases and it included different individual, family, school, peer, community, and society factors related to radicalization. Among family factors, variables such as parenting styles, significant others who do not use violence, and owning a house were identified as protective factors. Although this systematic review provided valuable information on the topic, family‐related search terms were not included in literature searches. Moreover, family‐related risk factors, consequences, and interventions were not reviewed. Thus, the current review differs from Lösel et al. (2018) as it also includes risk factors, consequences, interventions, and specific search terms that could locate all the studies specifically focused on family and radicalization.

A Campbell Collaboration registered protocol focused on putative risk and protective factors for cognitive and behavioral radicalization (Wolfowicz et al., 2020). Some results regarding putative factors for radicalization were recently published by Wolfowicz et al. (2019). It was found that parental involvement was a protective factor against radical attitudes and behaviors and being married was protective against radical attitudes. Whilst the systematic review conducted by Wolfowicz et al. (2019) represents an important contribution providing a better understanding of radicalization, the current review will specifically focus on family, including specific searches including family‐related risk and protective factors, consequences of radicalization for families, and family‐related interventions against radicalization. The current review differs from Wolfowicz et al. (2019) as it also includes protective factors, consequences, interventions, and specific family‐related search terms that will result in locating additional studies specifically focused on families.

Thus, there are no existing or registered systematic reviews specifically focused on family and radicalization, including specific family‐related keywords, searches, inclusion and exclusion criteria, coding and analyses. None of the previous reviews focused on both risk and protective factors, included family‐related consequences, and/or family‐related interventions. Our systematic review will address important gaps in the literature providing a global panorama regarding family factors and family‐related interventions and radicalization, including extensive literature searches specifically focused on family and radicalization, and make solid inferences about what works best against radicalization.

There are increasing efforts to describe, understand and decrease radicalization globally. Addressing radicalization and eliminating terrorism are among the most important national and international policy priorities. Although different groups become important in adolescence and adulthood, including peers, coworkers, and other social networks, families are a crucial part of the social capital of individuals throughout their lives (Coleman, 1988; Hoffmann & Dufur, 2018). Thus, our systematic review will provide insights and contribute to a bigger picture regarding radicalization, making it possible to improve evidence‐based policy and practice.

Having a global vision and comprehensive understanding of family‐related factors and interventions will make it possible to decrease risks and increase protective factors which will potentially reduce radicalization together with its detrimental consequences. After analyzing which family‐based programs and program components are effective to decrease radicalization, a new generation of prevention policy and practice can be designed, including components focused on the most important risk and protective factors, and consequences. Thus, policymakers will obtain valuable information that can be crucial for the design and development of a new generation policy and practice against radicalization which is not available yet. This systematic review will provide important information for evidence‐based policy and practice.

## 2 OBJECTIVES

This systematic review aims to answer the following research questions:

1. What are the family‐related risk and protective factors for radicalization?
2. What is the impact of radicalization on families?
3. To what extent are family‐based interventions against radicalization effective?

The review will answer these research questions by systematically gathering and synthesizing published and unpublished scientific literature on family‐related risk and protective factors for radicalization, the impact of radicalization on family, and studies that evaluate the impact of family‐based interventions on radicalization. Evidence permitting this review will also explore what components of family‐based interventions are most effective for countering radicalization. Thus, this systematic review will provide a global vision of scientific literature focused on family and radicalization including quantitative research.

## 3 METHODS

### 3.1 Criteria for considering studies for this review

#### 3.1.1 Types of studies

This systematic review will include quantitative studies focused on family‐related risk factors, protective factors, consequences, and interventions against radicalization. Empirical studies will be included, and reviews or editorial materials will be excluded. Both published and unpublished studies that follow the inclusion criteria will be included.

Quality of the included studies will be evaluated, although studies will not be excluded from the systematic review even if their quality is low. The systematic review will describe all the included studies pointing out their quality and to what extent results can be trusted. Low quality studies (i.e., other than randomized controlled trials and quasi‐experiments with a pretest, a posttest, an experimental and a control group in research on family‐related interventions) will be excluded from the meta‐analysis due to their high risk of bias. Nevertheless, single group studies with a pre‐ and posttest, or posttest only studies will be included in the systematic review (but not in the meta‐analysis). While single group designs are highly subject to bias, their inclusion can be justified if the area of research is newly emerging and there is lower likelihood of locating more rigorous studies. This is the case of research focused on family and radicalization.

##### Risk and protective factors

Studies will be included if they provide empirical data on the relation between any possible family‐related risk factor, protective factor, and radicalization. Family‐related factors and radicalization (including attitudes and behaviors) need to be explicitly measured. These factors need to focus on family structure, attitudes and behaviors of family members, or interpersonal relations within families. These can be correlational studies, or studies that compare groups with and without the risk or protective factor. Both bivariate and multivariate results will be included to discover direct and unique relations after controlling for covariates. Thus, studies will typically include analyses such as two by two contingency tables (and associated phi coefficients), means on a scaled variable for each category of a dichotomous variable (and associated point‐biserial correlation), correlation between two scaled variables (e.g., Pearson's correlation), and regression analyses. In cross‐sectional studies, risk and protective factors will be defined on a theoretical basis and treated as independent variables, whereas radicalization will be treated as a dependent variable. In longitudinal studies, risk and protective factors will precede radicalization.

Study designs focused on family‐related risk and protective factors will be cross‐sectional and preferably longitudinal. Although cross‐sectional designs are weaker than longitudinal designs, it is common to study risk factors for different problem behaviors through cross‐sectional studies on a theoretical basis. If possible, a moderator analysis will be run to assess the impact of cross‐sectional versus longitudinal designs.

##### Family impact of radicalization

Studies will be included if they provide empirical data on the impact of radicalization on family and family environment. Family‐related consequences of radicalization need to be explicitly measured. These can be correlational studies, or studies that compare groups including radicalized versus nonradicalized individuals in relation to family‐related consequences. Study designs focused on family consequences of radicalization will also be cross‐sectional and preferably longitudinal.

In cross‐sectional studies, family‐related consequences will be defined on a theoretical basis and treated as dependent variables, whereas radicalization will be treated as an independent variable. In longitudinal studies, radicalization will precede the family impact.

##### Family‐focused interventions

Intervention programs will be included if they are based on a randomized controlled trial where participants are randomly assigned to experimental (intervention) or control conditions (without an intervention) or a quasi‐experiment with a robust design (nonrandomized experimental vs. control group including a pretest and a posttest measures, and matched designs). One group pretest posttest intervention studies will be described in the systematic review but they will not be used for the meta‐analysis.

#### 3.1.2 Types of participants

##### Risk and protective factors

The systematic review will include international research conducted with any type of population if family‐related risk and protective factors, and radicalization in at least one family member are measured. Family refers to members by consanguinity (i.e., mother, father, children, siblings, cousins, aunts, uncles, and grandparents), and family members by marriage (i.e., husbands, wives, and long‐term partners). There will be no restrictions regarding study location or any characteristic of the participants. Thus, the review will include participants of any age, gender, ethnicity, socioeconomic status, and family structure. Populations from any part of the world, including low‐middle‐ and high‐income countries are going to be included.

##### Family impact of radicalization

This systematic review will not be restricted to any specific study location or any characteristic of the participants regarding family impact of radicalization if radicalization is measured in at least one family member together with its impact on at least one family member. Thus, this systematic review will include participants of any age, gender, ethnicity, socioeconomic status, and family structure. Also, populations from any part of the world, including low‐middle‐ and high‐income countries are going to be included. Again, family refers to members by consanguinity and marriage.

##### Family‐focused interventions

For family‐related interventions, this systematic review will include any type of population with at least one radicalized family member (by consanguinity and marriage) or at least one family member at‐risk of radicalization. Again, no restrictions regarding study location or characteristics of the participants will be used. Participants of any age, gender, ethnicity, socioeconomic status, and family structure, from any part of the world, will be included.

#### 3.1.3 Types of risk and protective factors

The systematic review will include any study that aims to discover the relation between family‐related factors and radicalization. These factors can be conceptualized as risk or protective factors for radicalization. Each of these studies needs to include a specific measure of a family‐related factor and a specific measure of radicalization of attitudes or behaviors.

A family‐related risk factor is defined as any factor related to families that is hypothesized to increase the risk of radicalization in a family member. A family‐related protective factor is defined as any factor related to families that is hypothesized to decrease the risk of radicalization in a family member. In longitudinal studies, these factors precede radicalization. In cross‐sectional studies, these factors are conceptualized as risk and protective factors, and they are treated as independent variables. Individuals or groups can be exposed to these factors at any moment of their lives. Age of exposure will be coded and used as a moderator, as it is expected that the importance of these factors could fluctuate through lifespan. Family‐related factors are variables that describe relationship styles, bonding, and circumstances within families. These can include, but are not limited to, parenting styles, marital status, divorce, parental behavioral problems, involvement, and unemployment.

#### 3.1.4 Types of variables associated with family impacts

This systematic review will include any study that measures the impact of radicalization on family members of radicalized individuals. These studies need to explicitly measure radicalization of attitudes and behavior and family‐related consequences. A family‐related consequence is defined as any variable that is hypothesized to be an undesirable consequence of radicalization for family members. In longitudinal studies, radicalization needs to be measured first, and consequences need to be measured afterward. In cross‐sectional studies, consequences are conceptualized on a theoretical basis and treated as dependent variables.

#### 3.1.5 Types of interventions

This systematic review will include any intervention that aims to modify family‐related factors to decrease cognitive or behavioral radicalization. Specifically, included interventions:

1. Include family‐related risk or protective factors among the intervention components, or
2. Families are the recipients of the interventions, or
3. Include specific components to prevent or buffer family‐related consequences.

These interventions could include, for example, family counseling and individual or group interventions focused on family‐related risk and protective factors for radicalization. Any intervention modality could be included, such as individual or group, face‐to‐face and online, manualized and unstructured interventions. Different types of programs including therapeutic, educational, and other types will be included. Programs could be implemented by researchers, educators, independent program developers and other providers. Interventions are going to be focused on both family members and family‐related risk and protective factors. Interventions that include a family component combined with other components that are not relevant to this systematic review will be included in the systematic review, but they will be excluded from the meta‐analysis if they do not provide a specific evaluation of the family component. If possible, moderator analyses will be performed to check if these different characteristics of the interventions influence the results.

#### 3.1.6 Types of outcome measures

##### Primary outcomes

###### Risk and protective factors and family‐focused interventions

This systematic review includes a broad range of outcomes related to radicalization. These outcomes focus on radicalization, radicalization to violence, extremism and terrorism including their different types such as right‐wing, left‐wing, religious extremism, and any other type of radicalization or extremism measured in the primary studies.

Primary outcomes included in this systematic review are extremism, violent extremism, radicalization, radicalization to violence and terrorism. Although these terms are frequently used interchangeably, there are certain differences regarding their definitions:

1. Extremism is defined as ideas that are opposed to mainstream social values.
2. Violent extremism is defined as beliefs and actions of individuals who engage in violent acts or support the use of violence to achieve goals related to their extreme ideas (Canada Centre for Community Engagement and Prevention of Violence, 2018).
3. Radicalization is a process through which individuals support and engage in activities that violate social norms shared by other members of the society (Kruglanski et al., 2014). Radicalization has also been defined as a process of adopting extreme views that differ from the mainstream beliefs and have a strong ideological basis (Bartlett & Miller, 2012). Although these beliefs per se are not necessarily a threat, according to the European Commission (2005a), they could lead to terrorism.
4. Radicalization to violence is defined as a process through which people acquire a series of beliefs, attitudes and ideologies, justifying the use of violence to achieve social goals and promote their ideas (Doosje et al., 2016). Radicalization to violence can include a cognitive component defined as attitudes and ideas that support violence as a means to promote these radical ideas, also including intentions to perpetrate these acts of violence. It also includes a behavioral component which consists of committing acts of extremist violence to promote radical ideas.
5. Terrorism has been defined as a commission of a terrorist act or joining a group to contribute to terrorist offences (European Commission, 2005b).

Based on the two‐pyramids model (McCauley & Moskalenko, 2017), outcomes will be measured taking into account both radicalization of opinion and radicalization of behavior. Radical and extremist attitudes are expected to be measured mostly with self‐reported and other‐reported measures such as questionnaires. Radical behaviors are expected to be measured also with self‐ and other‐reported measured, but they could also be measured with official records of radical behaviors including violent extremism and terrorist acts.

###### Family impact of radicalization

Consequences of radicalization for families will also be studied as a primary outcome of interest. These outcomes will focus on any negative impact of radicalization on families that could include, but will not be limited to, social exclusion, broken families, negative impact on wellbeing, mental health issues, and so forth. These outcomes will be measured taking into account both self‐reported and other‐reported measures regarding family‐related impact of radicalization. In longitudinal studies, radicalization will be measured first and these negative consequences will be measured afterward. In cross‐sectional studies, consequences will be defined on a theoretical basis and they will be treated as dependent variables. Family impact of radicalization is expected to be measured mostly with self‐reports and other‐reports measures such as questionnaires.

##### Secondary outcomes

Based on the located studies and their empirical findings, some secondary outcomes used as proxies of primary outcomes could be included in this systematic review. For studies focused on family‐related risk and protective factors, and interventions, secondary outcomes could include perceived group threat and in‐group superiority and hostility toward the out‐group. More possible secondary outcomes could be determined based on the located studies.

#### 3.1.7 Duration of follow‐up in family‐focused interventions

Studies reporting any duration of follow‐ups are eligible for inclusion. Studies will be grouped according to the duration of the follow‐up periods in categories such as: studies with a short follow‐up (0–3 months), studies with a medium follow‐up (between 3 and 6 months), and studies with a long‐term follow‐up (more than 6 months).

#### 3.1.8 Types of settings

There will be no search limitations regarding the year, language or geographical area. Thus, studies that meet the inclusion criteria will be included regardless of their settings. Searches will be conducted in English, but studies in any language will be included if located. Google Translate will be used for languages not understood by the authors of this systematic review (other than English, Spanish, French, Italian, Polish, Portuguese, and Romanian).

### 3.2 Search methods for identification of studies

The systematic literature searches will be performed on an extensive range of search locations to ensure that published as well as unpublished studies are located.

*Electronic searches*

For the identification of eligible studies, we will search titles, abstracts, keywords and/or subject/indexing terms with a combination of search terms using the Boolean operators “AND” and “OR.” These terms will be combined with the following terms searched in titles, abstracts, keywords and/or subject/indexing terms:

radicali* OR terror* OR extremis* OR “lone wol*” OR lone‐wol* OR “foreign fighter*” OR “single issue” OR Jihad* OR Islamis* OR Salaf* OR left‐wing OR far‐left OR right‐wing OR far‐right OR neo‐nazi* OR communis* OR nationalis* OR supremacist OR anarch* OR indoctrinat*

AND

family OR families OR familial OR parent* OR siblin* OR brother* OR sister* OR father* OR mother* OR child* OR son* OR daughter* OR cousin* OR uncle* OR aunt* OR generation* OR maternal OR paternal OR grandparent*

AND

risk* OR protect* OR factor* OR correlat* OR relat* OR predict* OR caus* OR determina* OR consequenc* OR interven* OR evaluat* OR program* OR treat* OR prevent* OR experiment* OR “cross‐section*” OR longitudinal* OR regress*

Searches will be performed in English as the vast majority of scientific literature is written in English and most of the studies written in other languages can be located with English terms in international databases. Google Translate will be used if studies in languages not read by the authors (other than English, Spanish, French, Italian, Polish, Portuguese, and Romanian) are located.

Electronic searches for the identification of studies will be performed in different academic databases including Campbell Systematic Reviews and Cochrane Library for reviews to scan reference lists, Criminal Justice Abstracts (EBSCO), Google Scholar (searches in titles only combining radicalization related terms with family related terms), ProQuest Platform (including, APA PsycArticles®‎, APA PsycInfo®‎, Health & Medical Collection‎, MEDLINE®‎‎, Periodicals Archive Online‎, Periodicals Index Online‎, ProQuest Dissertations & Theses Global, Psychology Database‎, and Publicly Available Content Database‎), Sage Journals Online and Archive, ScienceDirect, SCOPUS, Taylor & Francis Online, Web of Science (including Core Collection, Current Contents Connects, Derwent Innovations Index, Korean Journal Database, Russian Science Citation Index, SciELO Citation Index), and Wiley Online Library.

For each search location, information such as search location, date of the search and exact search syntax used for that search location will be recorded and reported in the final review.

### 3.3 Searching other resources

We will also search for gray literature on the websites of different agencies and professional organizations which include studies focused on countering radicalization:

- Department of Homeland Security (https://www.dhs.gov/topic/preventing-terrorism)
- Global Centre on Cooperative Security (https://www.globalcenter.org/publications/)
- Global Terrorism Research Centre (http://artsonline.monash.edu.au/gtrec/publications/)
- Hedayah (https://www.hedayahcenter.org/programs/)
- Impact Europe (http://impacteurope.eu/)
- National Consortium for the Study of Terrorism and Responses to Terrorism (START, https://www.start.umd.edu/radicalization-and-deradicalization)
- National Criminal Justice Reference Service (https://www.ojp.gov/ncjrs/new-ojp-resources)
- Public Safety Canada (https://www.publicsafety.gc.ca/index-en.aspx)
- RAN (https://ec.europa.eu/home-affairs/what-we-do/networks/radicalisation_awareness_network_en)
- Radicalisation Research (https://www.radicalisationresearch.org/)
- Royal United Services Institute (RUSI, https://rusi.org/)
- Terrorism Research Centre (http://www.terrorism.org/)

To identify more eligible studies, references of the included studies and references of the previously published narrative and systematic reviews will be screened. Documents citing the included studies will also be screened in Google Scholar, Scopus, and Web of Science. Moreover, hand‐searches of 2020 and 2021 volumes of the following journals will be performed:

- *Behavioral Sciences of Terrorism and Political Aggression*
- *Critical Studies on Terrorism*
- *Dynamics of Asymmetric Conflict*
- *Journal of Policing, Intelligence and Counter Terrorism*
- *International Journal of Conflict and Violence*
- *Journal for Deradicalization*
- *Journal of Interpersonal Violence*
- *Perspectives on Terrorism*
- *Studies in Conflict & Terrorism*
- *Terrorism & Political Violence*

Authors of the located studies and other experts in the field will be contacted and asked to provide published and unpublished studies focused on family and radicalization.

### 3.4 Data collection and analysis

#### 3.4.1 Description of methods used in primary research

##### Risk and protective factors

Primary research includes a great variety of methods. Regarding studies focused on risk and protective family‐related factors for radicalization, designs are mostly correlational. Most of these studies are cross‐sectional and conceptualize risk and protective factors on a theoretical basis. It is also hoped to locate and include some longitudinal projects where risk and protective factors are measured first and radicalization is measured afterward.

##### Family impact of radicalization

Most of the studies focused on family‐related consequences of radicalization are expected to have correlational designs. It is expected that these studies are cross‐sectional and conceptualize family‐related consequences on a theoretical basis. Nevertheless, it is also hoped to locate and include some longitudinal projects where radicalization is measured first and family‐related consequences are measured afterward.

##### Family‐focused interventions

Quantitative family‐related intervention studies against radicalization are expected to be mostly experimental or quasi‐experimental. Experimental designs that use randomized controlled trials are the gold standard in psychological and criminological research, but they are difficult to conduct and, therefore, it is expected that most of the included studies are quasi‐experimental (e.g., without randomization). Ideally, studies will include a pretest, a posttest, an experimental and a control group. The methodological quality of each study will be evaluated and specified. Thus, low‐, medium‐, and high‐quality studies will be described as such, providing a comprehensive panorama on what is already done and published, and what needs to be done next.

#### 3.4.2 Criteria for determination of independent findings

This systematic review will include each independent study in each analysis only once. All relevant effect sizes will be coded, and the dependencies issue will be solved at the analysis stage by selecting independent subsets for each analysis. When there are multiple effect sizes relevant to a particular analysis, the effect sizes will be divided by outcome construct and then robust variance estimation will be applied to handle multiple effect sizes per study. If there are effect sizes available for the whole group and subgroups (e.g., males and females), only the effect size for the whole group will be used, although subgroups can be analyzed separately if they are included in moderator analyses.

At the analysis stage, effect size multiplicity will be dealt with by identifying and categorizing the types of multiplicities in the included studies. Then, a reductionist approach (i.e., only one effect size extracted from a primary study) will be used when only one effect size is relevant according to the objectives of this systematic review and an integrative approach where effect sizes are combined will be used when there is more than one conceptually similar effect size per study (López‐López et al., 2018).

Studies in the field can sometimes report multiple and similar variables using the same sample (e.g., maternal warmth in relation to radicalization and paternal warmth in relation to radicalization), data from the same project can be published in multiple documents (e.g., a doctoral thesis and a journal article, two articles reporting on different variables from the same project and participants), and data from one project can be reported taking into account different subsamples in the same paper or in different papers (e.g., males and females, younger and older participants).

When multiple outcomes or predictors are reported in the same paper, their identification is straightforward. These will be reported as one study in the systematic review and combined in the meta‐analyses to one effect per project. Studies published by the same authors and studies with some overlap in research teams (or specific projects) will be analyzed as possibly reporting findings that are not independent. When these findings are reported in different documents, methodology sections will be thoroughly analyzed to check if samples are the same. In case of doubts, study authors will be contacted and asked to provide information about the independence of the findings. If findings are not independent, only the most comprehensive and complete reports will be included. If two or more reports are complimentary, they will be described as one study in the systematic review and, for the meta‐analysis, results will be combined. Groups (e.g., males and females) or multiple outcomes (e.g., cognitive and behavioral radicalization) can be analyzed through a moderator analysis.

#### 3.4.3 Selection of studies

After comprehensive searches in electronic databases, references located in databases that allow exporting references easily to reference management tools will be imported into EndNote software (EBSCO, ProQuest Platform, SCOPUS, Sage Journals Online and Archive, ScienceDirect, Taylor & Francis Online, and Web of Science). References located in databases that do not allow direct exporting will be screened in each database (Campbell Systematic Reviews and Cochrane Library for reviews to scan reference lists, Google Scholar, and Wiley Online Library). Additional searches will be performed on the websites of different agencies together with hand‐searches of specialized journals. For all the located studies, titles will be screened first according to the inclusion and exclusion criteria. If a study is potentially eligible based on title, abstracts will be screened according to the inclusion and exclusion criteria. Finally, if a study is potentially eligible based on both title and abstract, full texts will be screened according to the inclusion and exclusion criteria. All these stages will be performed by two independent researchers and an agreement rate will be calculated. Any possible doubts and discrepancies will be solved through a careful analysis, discussion and consensus.

Regarding the EndNote database, any study that focuses on family and radicalization based on titles and abstracts will be potentially eligible and saved for a full text screening that will also be performed by the two authors of this review. Full texts will be screened according to the inclusion and exclusion criteria.

Additionally, authors of the located studies and other experts in the field will be contacted and asked to provide published and unpublished studies on the topic. When received, authors of this systematic review will check if they are already among the located studies. In case of receiving new records sent by the contacted experts, they will be screened for eligibility based on titles and abstracts. If eligible, they will be incorporated to the database and their full texts will be screened together with other located studies. Authors will keep track of all decisions to provide an accurate PRISMA flowchart in the final review.

#### 3.4.4 Data extraction and management

A coding sheet including all the relevant information from each study has been developed (see Appendix A). There are three similar coding sheets for the three parts of this systematic review, but each includes specific information that is relevant to each subtopic. Studies will be coded including studyinformation, methodology, quality assessment, and results (adjusted and unadjusted).

Details regarding specific information to be included in each category are shown in Appendix A. Each study will be coded by the two authors of this systematic review. Disagreements will be solved through discussion and consensus. An agreement rate between the two coders will be calculated.

In the case of locating studies where radicalization of family member X is a risk factor for radicalization of family member Y, radicalization will be treated as both, risk factor (radicalization of family member X) and consequence (radicalization of family member Y). These studies will be double coded and included in both, the systematic review of family risk and protective factors, and the systematic review of family‐related consequences.

#### 3.4.5 Assessment of risk of bias in included studies

This review will assess the risk of bias taking into account the quality of the included studies. Although it is not planned to exclude studies from the review based on quality, each project will be assessed, and the quality of each paper explicitly reported. If possible, a meta‐regression based on the quality of the studies will be performed. Although all the studies that meet the inclusion criteria will be included in the systematic review, low quality studies will be excluded from the meta‐analysis.

##### Risk and protective factors, and family impact of radicalization

Quality of the studies will be assessed through the Cambridge Quality Checklist designed by Murray et al. (2009). The checklist will be applied by both authors independently, differences will be discussed and resolved by consensus. Table 1 shows the details regarding the application of the quality criteria to the included studies.

**Table 1:** Quality assessment according to the Cambridge Quality Checklist

Criterion	Scoring
Sampling	Studies including the whole population or a random sample are rated as high quality (1) and studies with convenience sampling or case‐control design are rated as low quality (0)
Response rates	Response rates above 70% and attrition below 10% are rated as high quality (1), response rates below 70% and attrition above 10% are rated as low quality (0)
Sample size	Studies with a sample size above 400 are rated as high quality (1), studies with a sample size below 400 are rated as low quality (0)
Measure of radicalization	Measures with reliability coefficients above 0.75 and face validity or convergent validity above 0.30 or more than one instrument are rated as high quality (1), other measures as low quality (0)
Measure of family factors	Measures with reliability coefficients above 0.75 and face validity or convergent validity above 0.30 or more than one instrument are rated as high quality (1), other measures as low quality (0)
Methodology used for studying risk/protective factor	Cross‐sectional designs are scored 1, retrospective designs are scored 2 and longitudinal prospective designs are scored as 3
Causal risk/protective factors	Studies with no comparison group and analysis of change are scored 1, studies with a comparison group but no control of confounders or change are scored 2, studies with no comparison group but a measure of change are scored 3, studies with a comparison group and a measure of change but no control of confounders are scored 4, studies with a comparison group statistically balanced or matched on confounders are scored 5, controlled nonexperimental studies with a measure of within‐individual change are scored 6 and randomized controlled trials are scored 7

##### Family‐focused interventions

Risk of bias in randomized trials will be assessed through RoB 2 tool (Sterne et al., 2019). RoB 2 focuses on domains such as trial design, implementation and reporting which are assessed through so called “signaling questions” that indicate the risk of bias. Based on the answers to these “signaling questions,” an algorithm indicates if the risk of bias is low, high or with “some concern.” There are three different versions of RoB 2 including a version for individually‐randomized parallel‐group trials, a test version for cluster‐randomized trials, and a test version for crossover trials. A RoB 2 version will be chosen based on the design of the included studies.

Risk of bias in nonrandomized intervention studies will be assessed through ROBINS‐I tool (Sterne et al., 2016). ROBINS‐I includes a series of questions focused on different possible types of bias in nonrandomized interventions. There are seven domains of potential bias that are assessed through “signalling questions.” The risk of bias judgment varies from low to critical risk. Risk of bias in family‐focused intervention studies will be assessed by both authors of this review independently. Again, discrepancies will be solved by discussion and consensus.

#### 3.4.6 Measures of effect

Some primary studies may provide unadjusted univariate coefficients that show direct relations such as correlations. Other primary studies may provide adjusted multivariate coefficients that control for confounders such as statistics derived from regression models. Some primary studies may provide both adjusted and unadjusted coefficients. Although there is a debate in the field on whether unadjusted or adjusted coefficients are the most appropriate for meta‐analyses (Hunter & Schmidt, 2004) both have advantages and disadvantages. Thus, the current meta‐analysis will run two separate types of analyses providing results for direct relations among variables and for relations after adjusting for confounders.

##### Risk and protective factors, and family impact of radicalization

Statistics needed to calculate the effect sizes will be extracted from primary studies. For unadjusted analyses, these statistics will mostly include coefficients such as Pearson's *r*, means, SDs, and the number of participants. For adjusted analyses, statistics will mostly include Bs, SEs, *β*s, and sample sizes. If statistics necessary to calculate effect sizes are unavailable in the published studies, authors will be contacted and asked to provide all the details necessary for the meta‐analysis.

All the effect size calculations will be performed using Comprehensive Meta‐Analysis Software (Borenstein et al., 2006). The statistic used for the calculation of the overall effect sizes will be chosen based on the primary studies taking into account the most frequently used coefficients. If most of the studies use correlation coefficients, standardized *r* using the Fisher's Z‐r transformation and variance will be used as the effect size. Other statistics expected to be found among the primary studies such as means, SDs, and sample sizes commonly used in group comparisons will be transformed into *r* (or other most commonly used coefficient among the primary studies). Transformations will be done using Comprehensive Meta‐Analysis Software based on formulas by Lipsey and Wilson (2001).

Conversion from *d* to *r* will be done as follows:

$$r=\frac{d}{\sqrt{d^{2}+a}}.$$

$$a=\frac{\left(n_{1}+n_{2}\right)2}{n_{1}n_{2}}.$$

For the calculation of adjusted effect sizes, the standardized regression coefficient will be used together with its associated SE as the basis for the inverse variance weight rather than the weight based on sample size.

Where the independent variable is dichotomous (e.g., divorced parents), effect sizes will be calculated as:

$$d=\frac{B}{SD\left(\mathrm{dependent}\mathrm{variable}\right)}$$

where B is the unstandardized regression coefficient from an OLS regression model (not from other model types).

For logistic regression with dichotomous independent variables, the following equation will be used (Lipsey & Wilson, 2001, p. 202):

d=ln(OR).5513,

where the OR is the partial OR in the model or exp(B).

An *r* above 0 indicates that higher scores in protective factors, risk factors or consequences are related to high scores in radicalization. It also indicates desirable treatment effects in intervention studies. An *r* below 0 indicates that low scores in protective factors, risk factors or consequences are related to high scores in radicalization. An *r* of 0 (or confidence intervals that include 0) indicates insufficient evidence of relation between variables.

##### Family‐focused interventions

The meta‐analysis on family‐focused interventions will be performed using effect sizes extracted from the included primary studies. For unadjusted analyses, these statistics will likely include pre‐ and posttest means, SDs, and number of participants in the intervention and control groups. These statistics can also include outcome frequencies by intervention versus control groups, gain scores, and so forth. Adjusted analyses are uncommon in intervention studies in the field, but they will meta‐analyzed separately if available using the same equations as those used for the calculation of effect sizes for risk and protective factors, and consequences.

Again, the statistic used for the calculation of the overall effect sizes will be chosen taking into account the most frequently used coefficients in the primary studies. It is expected that the most appropriate effect size is Cohen's *d*. In such case, other statistics will be transformed into *d* using Comprehensive Meta‐Analysis Software based on formulas by Lipsey and Wilson (2001). A *d* above 0 indicates a desirable effect of an intervention (a decrease in radicalization or risk factors that is bigger in the experimental group in comparison to the control group). A *d* below 0 indicates an undesirable effect of the intervention (a decrease in radicalization or risk factors that is bigger in the control group in comparison to the experimental group). A *d* of 0 (or confidence intervals that include 0) indicates insufficient evidence of a treatment effect.

#### 3.4.7 Unit of analysis issues

Results based on each independent sample will be used as the unit of analysis. Unit‐of‐analysis issues may refer to issues regarding clustering, crossover designs, and studies with multiple outcome measurement time‐points. In cluster‐trials, groups of individuals are allocated to treatment versus control conditions (Higgins et al., 2020). In some studies, research participants may be grouped, for example, in prisons or schools. This clustering is an issue because there are similarities among observations form the same cluster and these observations are usually not independent (Hedges & Rhoads, 2011). These studies could have artificially small SEs and their weights could be artificially inflated if they are included in a meta‐analysis without a correction for clustering (Higgins et al., 2020). To solve these issues, corrections for clustering in SEs of the effect sizes are necessary (Hedges, 2007). Thus, the variance will be increased by [1 + (*n* – 1) × ICC], where *n* = number of individuals in a cluster and ICC is the intra‐class correlation. If studies do not provide an ICC, following Armstrong et al. (2017), an ICC of 0.03 will be assumed and sensitivity analyses with an ICC of 0, 0.01, 0.02, and 0.03 will be performed.

Crossover designs are studies in which participants are assigned to a sequence of interventions. For example, participants can be assigned to intervention A or B, and then participants who received intervention A receive the intervention B while participants who received the intervention B receive the intervention A (Higgins et al., 2020). Crossover trials will be included if an intervention condition is compared to a control condition that does not receive an intervention. It is expected that most of the included projects are parallel studies and locating crossover designs is unlikely. If crossover designs are located, only data from the first period of crossover trials will be included to make them as similar as possible to other included studies. This approach will avoid carry‐over problems.

We do not expect to find studies with multiple outcome measurement time‐points, although some trials may include posttests and follow‐ups. In such cases, posttests will be included in one analysis together with the remaining studies, and separate meta‐analyses will be conducted for the follow‐ups.

#### 3.4.8 Dealing with missing data

Some primary studies may have missing data, such as including only descriptions of the results without including the statistics necessary to calculate effect sizes. It is also possible to find studies that include incomplete information with some statistics that are insufficient for the calculation of the effect sizes (e.g., means without SDs or other coefficients that could be used for the calculation of the effect size). It is common to find studies that only provide significant results and do not provide the coefficients if they are nonsignificant (e.g., only marking them as ns). In all these cases, authors of the primary studies will be contacted and asked to provide the missing results or the databases for their calculation.

If authors of the primary studies do not respond or do not provide the missing results, studies that only include descriptions of the findings without numerical results and studies that include incomplete information that is insufficient for the calculation of the effect sizes will be excluded from statistical analyses. Studies that provide information for the calculation of the effect sizes of the significant results but do not provide information for the calculation of the effect sizes of the nonsignificant results will be included. The direction and the sample sizes are usually known and, following Wilson et al. (2001), it will be assumed that the effect is between zero and the smallest significant effect size. This means that the midpoint value is used. Sensitivity analyses will be run to confirm that the weighted effect size is not substantially affected by this imputation.

#### 3.4.9 Assessment of reporting biases

Publication bias will be assessed through trim and fill analysis focused on the funnel plot asymmetry that is identified and corrected for. In trim and fill, the small studies that cause asymmetry in the funnel plot are “trimmed,” the center of the funnel plot is estimated, and the missing studies are “filled” around the center (Higgins et al., 2019). This method provides the number of missing studies and an adjusted effect size.

#### 3.4.10 Data synthesis

Data will be synthesized through a meta‐analysis conducted with a Comprehensive Meta‐Analysis software. Analyses will be based on Lipsey and Wilson (2001). Separate meta‐analyses will be conducted for different family related risk and protective factors, family‐related outcomes and interventions. After a thorough analysis of the primary studies, variables that are theoretically similar could be grouped in categories for the meta‐analyses. At least two primary effect sizes will be required to perform each meta‐analysis. Statistics needed for the meta‐analysis will be extracted from primary studies and entered in the software. Then, statistics from each individual study will be transformed to a common effect size that will be chosen based on the most commonly used analyses among the included studies. An overall effect size will be calculated for each risk factor, protective factor, consequence, and intervention if at least two studies are available. The random effects method will be used for data synthesis as the studies are expected to be heterogeneous.

#### 3.4.11 Subgroup analysis and investigation of heterogeneity

Cochran's *Q* and *I* ^2^ will be used to assess heterogeneity. Although *Q* is the traditional test to assess heterogeneity in meta‐analyses, it can be underpowered when the number of studies included in a meta‐analysis is low. Thus, *I* ^2^ will also be calculated to assess heterogeneity. *I* ^2^ shows whether the variability across studies can be attributed to real differences or to chance (Higgins et al., 2003). The test of heterogeneity with *p* value <.10 combined with an *I* ^2^ value of 30% or greater shows evidence of heterogeneity across studies (Higgins et al., 2019).

Subgroups will be analyzed through meta‐regression techniques. For all the three objectives of this systematic review, some potential subgroup comparisons include gender, age groups, locations and contexts. This is mainly because risk and protective factors and consequences can differ among these groups and interventions can also impact differently males and females, younger and older participants, participants in different location (e.g., geographic areas or countries) and their impact can be different depending on a context (e.g., prisons, schools).

Other potential moderators include the quality of the included studies assessed according to the Cambridge Quality Checklist, RoB 2, and ROBINS‐I. For example, in correlational research, studies with smaller samples can be compared to studies with bigger samples. For intervention studies, quasi‐experimental projects may be compared to randomized controlled trials. Given that randomized controlled trials are considered the gold standard for evaluating the effectiveness of interventions, their results will be considered as the most reliable compared to other research designs. The final review will distinguish between a priori and exploratory analyses.

#### 3.4.12 Sensitivity analysis

Sensitivity analyses will be performed if missing data are imputed as explained in “dealing with missing data” section. Sensitivity analyses will also be performed if correction for clustering is done for the studies that do not provide an ICC, as stated in “unit of analysis issues.” Additional sensitivity analyses will be run to discover if the results of the meta‐analysis were influenced by the risk of bias of the included studies. No other sensitivity analyses are planned to be performed.

#### 3.4.13 Treatment of qualitative research

Qualitative research will not be included in this review.

## CONTRIBUTIONS OF AUTHORS

This systematic review will be conducted by a team from University of Cordoba (Spain).

- *Content*: Izabela Zych and Elena Nasaescu.
- *Systematic review methods*: Izabela Zych and Elena Nasaescu.
- *Statistical analysis*: Izabela Zych and Elena Nasaescu.
- *Information retrieval*: Izabela Zych and Elena Nasaescu.

Izabela Zych is an Associate Professor in Psychology at the University of Cordoba and has broad experience and expertise in conducting systematic reviews and meta‐analyses focused on different topics related to violence. She has successfully led and published similar studies, including papers in the leading journals in the field. She is a member of Campbell Collaboration Crime and Justice Group Steering Committee including the Terrorism and Security subgroup.

Elena Nasaescu is a PhD candidate supervised by Izabela Zych, who is currently in the final stage of her PhD. She has a specific training in conducting systematic reviews and meta‐analyses, and excellent methodological skills.

## DECLARATIONS OF INTEREST

The authors declare that there are no conflicts of interests. However, one of the review authors has an internal role within the Campbell Collaboration Crime and Justice Group. Specifically, Izabela Zych is a member of the Campbell Collaboration Crime and Justice Group Steering Committee, including the Campbell/DHS Counter‐Terrorism Systematic Review Advisory Board. Thus, Izabela Zych will not be involved in any editorial or internal Campbell Collaboration communications about this review.

## PRELIMINARY TIMEFRAME

We plan to submit a draft of the systematic review before December 31, 2021.

## PLANS FOR UPDATING THIS REVIEW

We plan to produce an updated version of this review every four years. The lead author (Izabela Zych) will be in charge of coordinating the revised versions.

## APPENDIX A CODING SCHEME

**RISK AND PROTECTIVE FACTORS FOR RADICALIZATION**

1. **Coder and coding date:**
2. **Study information**
  a) Study ID
  b) Authors
  c) Publication year
  d) Study title
  e) Document language
  f) Document type: journal article, PhD thesis, book, book chapter, agency/government report, unpublished document, other (specify)
  g) Country where the study was conducted
  h) Year in which study was conducted
  i) Funding agency type: Local university, local administration, country government, European Union, NGO, private company, other (specify).
  j) Funding agency name
  k) Conflict of interest: yes/no (if yes, describe)
3. **Methodology**

**3.1. Participants (if longitudinal, specify for each wave)**

a) Sample size
b) Age
c) Sex: percentage of male, female, other (specify)
d) Ethnic‐cultural background: percentage of Caucasian, African American, Latin American, Asian American, other (specify)
e) Socioeconomic status: low, medium, high, mixed
f) Recruitment strategy: convenience sampling (specify sampling details), random selection
  from a population, other (specify)
g) Settings where participants were recruited (e.g., prisons, schools)
h) Attrition and response rate

**3.2. Design and procedure**

a) List of risk and protective factors measured and definitions of each risk and protective factor (constructs)
b) Age of exposure to each risk and protective factor and age of radicalization
c) Instruments used to measure each risk/protective factor (authors, number of items, response scale and psychometric properties)
d) Source of risk and protective factor measure (for each factor): self‐reports, other‐reports (specify), official records, other (specify)
e) Terms used to describe radicalization (radicalization, radicalization to violence, extremism, violent extremism, terrorism, other‐specify) and definition of radicalization used by the authors
f) Instruments used to measure radicalization (authors, number of items, response scale and psychometric properties)
g) Source of radicalization measure: self‐reports, other‐reports (specify), official records, other (specify)
h) Design: cross‐sectional, longitudinal, other (describe)
i) If longitudinal, specify: number of follow‐ups, duration of follow‐up (in years), time period between each wave, waves at which risk/protective factors were measured, waves at which radicalization was measured
j) Procedure for data collection: who collected the data, individual or group collection, context for data collection (e.g., prison, school, other – specify)
k) Ethics committee approval of the study: yes/no (if yes, specify the committee)

4. **Quality assessment according to Cambridge Quality Checklist including the following scores (see** Table 1 **):**

a) Sampling: 0/1
b) Response rates: 0/1
c) Sample size: 0/1
d) Measure of correlate: 0/1
e) Measure of outcome: 0/1
f) Risk factor: 1, 2 or 3
g) Causal risk factor: 1 to 7

5. **Results**
  5.1. Unadjusted results
  a. Correlation: yes/no, if yes specify for each study variable:
    i. Correlation coefficient: Pearson, Spearman, Point‐biserial, other (specify)
    ii. Coefficient value, direction and p value
    iii. Number of participants used for the calculation of the coefficient
    iv. Narrative description of the result: variable related to more radicalization, variable related to less radicalization, no relation found
  b. Mean comparison: yes/no, if yes, specify for each study variable:
    i. Mean and standard deviation in radicalized and nonradicalized groups
    ii. Test and coefficient used for mean comparison: ANOVA, Student's t, Cohen's d, other (specify)
    iii. Coefficient value, direction (if applicable), degrees of freedom (if applicable), confidence intervals (if applicable), standard error (if applicable) and p value
    iv. Number of participants in radicalized and nonradicalized groups used for the comparison
    v. Narrative description of the results: variable related to more radicalization, variable related to less radicalization, no relation found
  c. Frequencies/proportions: yes/no, if yes, specify for each study variable:
    i. Number and proportion of radicalized individuals with the predictor
    ii. Number and proportion of nonradicalized individuals with the predictor
    iii. Test and coefficient used for frequencies/proportions comparison: chi‐square, odds ratios, phi, other (specify)
    iv. Coefficient value, direction (if applicable), degrees of freedom (if applicable), confidence intervals (if applicable), standard error (if applicable) and p value
    v. Narrative description of the results: variable related to more radicalization, variable related to less radicalization, no relation found
  d. Other unadjusted tests and coefficients (if applicable), including numbers of participants used for their calculation, coefficient values, coefficient directions (if applicable), standard errors (if applicable), confidence intervals (if applicable), degrees of freedom (if applicable), and other relevant information (specify)
  e. Calculated effect size, confidence intervals and standard error
  f. Other relevant information (if applicable)
  5.2. Adjusted results
  a. Linear regression: yes/no, if yes, specify for each predictor:
    i. Unstandardized (B) and standardized regression (beta) coefficients values
    ii. Coefficient direction and p value
    iii. Standard error
    iv. Standard deviation of the dependent variable
    v. Number of participants used for the calculation of the coefficient
    vi. Variables controlled for
    vii. Narrative description of the result: variable related to more radicalization, variable related to less radicalization, no relation found
  b. Logistic regression: yes/no, if yes, specify for each predictor:
    i. Odds Ratio/Exp(B)
    ii. Coefficient direction and p value
    iii. Standard error
    iv. Confidence intervals
    v. Number of radicalized and nonradicalized participants used for the calculation of the coefficient
    vi. Variables controlled for
    vii. Narrative description of the result: variable related to more radicalization, variable related to less radicalization, no relation found
  c. Other adjusted tests and coefficients (if applicable), including numbers of participants used for their calculation, coefficients values, coefficient directions (if applicable), standard errors (if applicable), confidence intervals (if applicable), degrees of freedom (if applicable), variables controlled for, and other relevant information (specify)

d. Calculated effect size, confidence intervals and standard error
e. Other relevant information (if applicable)

**IMPACT OF RADICALIZATION ON FAMILIES**

1. **Coder and coding date:**
2. **Study information**
  a) Study ID
  b) Authors
  c) Publication year
  d) Study title
  e) Document language
  f) Document type: journal article, PhD thesis, book, book chapter, agency/government report, unpublished document, other (specify)
  g) Country where the study was conducted
  h) Year in which study was conducted
  i) Funding agency type: Local university, local administration, country government, European Union, NGO, private company, other (specify).
  j) Funding agency name
  k) Conflict of interest: yes/no (if yes, describe)
3. **Methodology**
  **3.1. Participants (if longitudinal, specify for each wave)**
  a) Sample size
  b) Age
  c) Sex: percentage of male, female, other (specify)
  d) Ethnic‐cultural background: percentage of Caucasian, African American, Latin American, Asian American, other (specify)
  e) Socioeconomic status: low, medium, high, mixed
  f) Recruitment strategy: convenience sampling (specify sampling details), random selection from a population, other (specify)
  g) Settings where participants were recruited (e.g., prisons, schools)
  h) Attrition and response rate
  **3.2. Design and procedure**
  a) List of consequences measured and definitions of each consequence (constructs)
  b) Age of exposure to radicalization and age when each consequence appeared
  c) Instruments used to measure each consequence (authors, number of items, response scale and psychometric properties)
  d) Source of consequences measure (for each factor): self‐reports, other‐reports (specify), official records, other (specify)
  e) Terms used to describe radicalization (radicalization, radicalization to violence, extremism, violent extremism, terrorism, other‐specify) and definition of radicalization used by the authors
  f) Instruments used to measure radicalization (authors, number of items, response scale and psychometric properties)
  g) Source of radicalization measure: self‐reports, other‐reports (specify), official records, other (specify)
  h) Design: cross‐sectional, longitudinal, other (describe)
  i) If longitudinal, specify: number of follow‐ups, duration of follow‐up (in years), time period between each wave, waves at which radicalization was measured, waves at which consequences were measured
  j) Procedure for data collection: who collected the data, individual or group collection, context for data collection (e.g., prison, school, other – specify)
  k) Ethics committee approval of the study: yes/no (if yes, specify the committee)
4. **Quality assessment according to Cambridge Quality Checklist including the following scores (see** Table 1 **):**
  a) Sampling: 0/1
  b) Response rates: 0/1
  c) Sample size_0/1
  d) Measure of correlate: 0/1
  e) Measure of outcome: 0/1
  f) Risk factor: 1, 2 or 3
  g) Causal risk factor: 1 to 7
5. **Results**
  5.1. Unadjusted results
  a) Correlation: yes/no, if yes specify for each study variable:
    i. Correlation coefficient: Pearson, Spearman, Point‐biserial, other (specify)
    ii. Coefficient value, direction and p value
    iii. Number of participants used for the calculation of the coefficient
    iv. Narrative description of the result: radicalization related to higher scores in a consequence, radicalization related to lower scores in a consequence, no relation found
  b) Mean comparison: yes/no, if yes, specify for each study variable:
    i. Mean and standard deviation in radicalized and nonradicalized groups
    ii. Test and coefficient used for mean comparison: ANOVA, Student's t, Cohen's d, other (specify)
    iii. Coefficient value, direction (if applicable), degrees of freedom (if applicable), confidence intervals (if applicable), standard error (if applicable) and p value
    iv. Number of participants in radicalized and nonradicalized groups used for the comparison
    v. Narrative description of the results: radicalization related to higher scores in a consequence, radicalization related to lower scores in a consequence, no relation found
  c) Frequencies/proportions: yes/no, if yes, specify for each study variable:
    i. Number and proportion of radicalized individuals with the consequence
    ii. Number and proportion of nonradicalized individuals with the consequence
    iii. Test and coefficient used for frequencies/proportions comparison: chi‐square, odds ratios, phi, other (specify)
    iv. Coefficient value, direction (if applicable), degrees of freedom (if applicable), confidence intervals (if applicable), standard error (if applicable) and p value
    v. Narrative description of the results: radicalization related to higher scores in a consequence, radicalization related to lower scores in a consequence, no relation found
  d) Other unadjusted tests and coefficients (if applicable), including numbers of participants used for their calculation, coefficients values, coefficient directions (if applicable), standard errors (if applicable), confidence intervals (if applicable), degrees of freedom (if applicable), and other relevant information (specify)
  e) Calculated effect size, confidence intervals and standard error
  f) Other relevant information (if applicable)
  5.2. Adjusted results
  a. Linear regression: yes/no, if yes, specify for each consequence:
    i. Unstandardized (B) and standardized regression (beta) coefficients values
    ii. Coefficient direction and p value
    iii. Standard error
    iv. Standard deviation of the dependent variable
    v. Number of participants used for the calculation of the coefficient
    vi. Variables controlled for
    vii. Narrative description of the result: radicalization related to higher scores in a consequence, radicalization related to lower scores in a consequence, no relation found
  b. Logistic regression: yes/no, if yes, specify for each predictor:
    i. Odds Ratio/Exp(B)
    ii. Coefficient direction and p value
    iii. Standard error
    iv. Confidence intervals
    v. Number of participants affected and not affected by the consequence used for the calculation of the coefficient
    vi. Variables controlled for
    vii. Narrative description of the result: radicalization related to higher scores in a consequence, radicalization related to lower scores in a consequence, no relation found
  c. Other adjusted tests and coefficients (if applicable), including numbers of participants used for their calculation, coefficient values, coefficient directions (if applicable), standard errors (if applicable), confidence intervals (if applicable), degrees of freedom (if applicable), and other relevant information (specify)
  d. Calculated effect size, confidence intervals and standard error
  e. Other relevant information (if applicable)

**FAMILY‐BASED INTERVENTIONS**

1. **Coder and coding date:**
2. **Study information**
  a) Study ID
  b) Authors
  c) Publication year
  d) Study title
  e) Document language
  f) Document type: journal article, PhD thesis, book, book chapter, agency/government report, unpublished document, other (specify)
  g) Country where the study was conducted
  h) Year in which study was conducted
  i) Funding agency type: Local university, local administration, country government, European Union, NGO, private company, other (specify).
  j) Funding agency name
  k) Conflict of interest: yes/no (if yes, describe)
3. **Methodology**
  **3.1. Participants (specify for pretest and posttest)**
  a) Sample size: total size, number of participants in the experimental group, number of participants in the control group
  b) Age
  c) Sex: percentage of male, female, other (specify)
  d) Ethnic‐cultural background: percentage of Caucasian, African American, Latin American, Asian American, other (specify)
  e) Socioeconomic status: low, medium, high, mixed
  f) Recruitment strategy: convenience sampling (specify sampling details), random selection from a population, other (specify)
  g) Settings where participants were recruited (e.g., prisons, schools)
  h) Attrition and response rate
  **3.2. Design and procedure**
  a) Design: randomized controlled trial, quasi‐experiment, other (specify)
  b) Assignment to experimental versus control group: random‐individuals, random‐clusters (specify), convenience (specify), other (specify)
  c) Radicalization measured at: pre‐ and posttest in experimental and control groups, pre‐ and posttest in experimental group only (no controls), posttest only in experimental and control groups, posttest in experimental group only (no controls), other (specify)
  d) Period between pretest and posttest
  e) Presence of follow‐up (yes/no), if yes, specify duration
  f) Terms used to describe radicalization (radicalization, radicalization to violence, extremism, violent extremism, terrorism, other‐specify) and definition of radicalization used by the authors
  g) Instruments used to measure radicalization (authors, number of items, response scale and psychometric properties)
  h) Source of radicalization measure: self‐reports, other‐reports (specify), official records, other (specify)
  i) Procedure for data collection: who collected the data, individual or group collection, context for data collection (e.g., prison, school, other – specify)
  j) Ethics committee approval of the study: yes/no (if yes, specify the committee)
4. **Intervention**
  a) Duration (days/months/years) and intensity (number of sessions)
  b) Topics/units (short description)
  c) Intervention strategies: cognitive‐behavioral, multisystemic therapy, counseling, other (specify)
  d) Target group: radicalized individuals, families, other (specify)
  e) Providers: researchers, practitioners within the context, independent companies, other (specify)
  f) Details of the intervention received by the control group: treatment as usual, waiting list, other (specify)
  g) other relevant information (specify)
5. **Quality assessment**
  a) Risk of bias in randomized trials according to RoB 2 tool
  b) Risk of bias in nonrandomized intervention studies according to ROBINS‐I tool
6. **Results**

**6.1. Unadjusted results**

a. Mean comparison: yes/no, if yes, specify for each study variable:
  i. Mean and standard deviation in radicalization in pre‐ and posttest for experimental and control groups
  ii. Test and coefficient used for mean comparison: Repeated measures ANOVA, ANCOVA, Paired Samples t‐test, other (specify)
  iii. Coefficient value, direction (if applicable), degrees of freedom (if applicable), confidence intervals (if applicable), standard error (if applicable) and p value
  iv. Number of participants in experimental and control group at pre‐ and posttest
  v.  Narrative description of the results: radicalization decreased, radicalization increased, no effect of intervention found
b. Frequencies/proportions: yes/no, if yes, specify for each study variable:
  i. Number and proportion of radicalized individuals in experimental and control group at pre‐ and posttest
  ii. Number and proportion of radicalized individuals in experimental and control group at pre‐ and posttest
  iii. Test and coefficient used for frequencies/proportions comparison: odds ratios, chi‐square, phi, other (specify)
  iv. Coefficient value, direction (if applicable), degrees of freedom (if applicable), confidence intervals (if applicable), standard error (if applicable) and p value
  v.  Narrative description of the results: radicalization decreased, radicalization increased, no effect of intervention found
c. Other unadjusted tests and coefficients (if applicable), including numbers of participants at pre‐ and posttest in the experimental and control group used for their calculation, coefficients values, coefficient directions (if applicable), standard errors (if applicable), confidence intervals (if applicable), degrees of freedom (if applicable), and other relevant information (specify)
d. Calculated effect size, its standard error and confidence intervals
e. Other relevant information (if applicable)
  **6.2. Adjusted results**
  a. Linear regression: yes/no, if yes, specify for each consequence:
    i. Unstandardized (B) and standardized regression (beta) coefficients values
    ii. Coefficient direction and p value
    iii. Standard error
    iv. Standard deviation of the dependent variable
    v. Number of participants used for the calculation of the coefficient
    vi. Variables controlled for
    vii. Narrative description of the result: radicalization related to higher scores in a consequence, radicalization related to lower scores in a consequence, no relation found
  b. Logistic regression: yes/no, if yes, specify for each predictor:
    i. Odds Ratio/Exp(B)
    ii. Coefficient direction and p value
    iii. Standard error
    iv. Confidence intervals
    v. Number of participants affected and not affected by the consequence used for the calculation of the coefficient
    vi. Variables controlled for
    vii. Narrative description of the result: radicalization decreased, radicalization increased, no effect of intervention found
  c. Other adjusted tests and coefficients (if applicable), including numbers of participants used for their calculation, coefficient values, coefficient directions (if applicable), standard errors (if applicable), confidence intervals (if applicable), degrees of freedom (if applicable), variables controlled for, and other relevant information (specify)
  d. Other relevant information (if applicable)

## References

[cl21190-bib-0001] Armstrong, E. , Eggins, E. , Reid, N. , Harnett, P. , & Dawe, S. (2017). Parenting interventions for incarcerated parents to improve parenting knowledge and skills, parent well‐being, and quality of the parent–child relationship: A systematic review and meta‐analysis. Journal of Experimental Criminology, 14, 279–317. 10.1007/s11292-017-9290-6

[cl21190-bib-0002] Bandura, A. (1991). Social cognitive theory of moral thought and action. In W. M. Kurtines , & J. L. Gewirtz (Eds.), Handbook of moral behavior and development (Vol. 1, pp. 45–103). Erlbaum.

[cl21190-bib-0003] Bartlett, J. , & Miller, C. (2012). The edge of violence: Towards telling the difference between violent and non‐violent radicalization. Terrorism and Political Violence, 24(1), 1–21.

[cl21190-bib-0004] Van Bergen, D. D. , Ersanilli, E. F. , Pels, T. V. M. , & De Ruyter, D. J. (2016). Turkish‐Dutch youths' attitude toward violence for defending the in‐group: What role does perceived parenting play? Peace and Conflict: Journal of Peace Psychology, 22(2), 120–133. 10.1037/pac0000173

[cl21190-bib-0005] Besemer, S. , Farrington, D. P. , & Bijleveld, C. C. (2017). Labeling and intergenerational transmission of crime: The interaction between criminal justice intervention and a convicted parent. PLOS One, 12, e0172419. 28273104 10.1371/journal.pone.0172419 PMC5342201

[cl21190-bib-0006] Borenstein, M. , Hedges, L. V. , Higgins, J. P. T. , & Rothstein, H. R. (2006). *Comprehensive meta‐analysis (Version 2.2.027) [Computer software]*. Biostat.

[cl21190-bib-0007] Borum, R. (2004). Psychology of terrorism. University of South Florida

[cl21190-bib-0008] Borum, R. (2012). Radicalization into violent extremism I: A review of social science theories. Journal of Strategic Security, 4(4), 7–36. 10.5038/1944-0472.4.4.1

[cl21190-bib-0009] Canada Centre for Community Engagement and Prevention of Violence . (2018). National strategy on countering radicalization to violence. Government of Canada.

[cl21190-bib-0010] Coleman, J. S. (1988). Social capital in the creation of human capital. The American journal of sociology, 94, S95–S120.

[cl21190-bib-0011] Creative Associates International . (n.d.). Tunisia—ETTYSAL . https://www.creativeassociatesinternational.com/past-projects/tunisia-ettysal/

[cl21190-bib-0012] Dhumad, S. , Candilis, P. J. , Cleary, S. D. , Dyer, A. R. , & Khalifa, N. (2020). Risk factors for terrorism: A comparison of family, childhood, and personality risk factors among Iraqi terrorists, murderers, and controls. Behavioral Sciences of Terrorism and Political Aggression, 12, 72–88. 10.1080/19434472.2019.1591481

[cl21190-bib-0013] Doosje, B. , Moghaddam, F. M. , Kruglanski, A. W. , De Wolf, A. , Mann, L. , & Feddes, A. R. (2016). Terrorism, radicalization, and de‐radicalization. Current Opinion in Psychology, 11, 79–84. 10.1016/j.copsyc.2016.06.008

[cl21190-bib-0014] Dugas, M. , & Kruglanski, A. W. (2014). The quest for significance model of radicalization: Implications for the management of terrorist detainees. Behavioral Sciences & The Law, 32, 423–439. 10.1002/bsl.2122 24802748

[cl21190-bib-0015] European Commission . (2005a). Council of Europe Convention on the Prevention of Terrorism. Warsaw: Council of Europe Treaty Series, No. 196.

[cl21190-bib-0016] European Commission . (2005b). The European Union counter‐terrorism strategy. Brussels: European Commission.

[cl21190-bib-0017] Farrington, D. P. , Coid, J. W. , & Murray, J. (2009). Family factors in the intergenerational transmission of offending. Criminal Behaviour and Mental Health, 19, 109–124. 19274628 10.1002/cbm.717

[cl21190-bib-0018] Feddes, A. R. , & Gallucci, M. (2015). A literature review on methodology used in evaluating effects of preventive and de‐radicalisation interventions. Journal for Deradicalization, 5, 1–27.

[cl21190-bib-0019] Gielen, A. J. (2015). Supporting families of foreign fighters: A realistic approach for measuring the effectiveness. Journal for Deradicalization, 1(2), 21–48.

[cl21190-bib-0020] Gottfredson, M. , & Hirschi, T. (2020). Modern control theory and the limits of criminal justice. Oxford University Press.

[cl21190-bib-0021] Guru, S. (2012). Under siege: Families of counter‐terrorism. British Journal of Social Work, 42(6), 1151–1173. 10.1093/bjsw/bcs089

[cl21190-bib-0022] Harris‐Hogan, S. (2014). The importance of family: The key to understanding the evolution of Jihadism in Australia. Security Challenges, 10(1), 31–49.

[cl21190-bib-0023] Hedges, L. V. (2007). Effect sizes in cluster‐randomized designs. Journal of Educational and Behavioral Statistics, 32, 341–370. 10.3102/1076998606298043

[cl21190-bib-0024] Hedges, L. V. , & Rhoads, C. H. (2011). Correcting an analysis of variance for clustering. British Journal of Mathematical and Statistical Psychology, 64, 20–37. 10.1111/j.2044-8317.2010.02005.x 21506943

[cl21190-bib-0025] Higgins, J. P. , Thomas, J. , Chandler, J. , Cumpston, M. , Li, T. , Page, M. J. & Welch, V. A. , (Eds.). (2019). Cochrane handbook for systematic reviews of interventions. John Wiley & Sons. 10.1002/14651858.ED000142 PMC10284251 31643080

[cl21190-bib-0027] Higgins, J. P. , Thompson, S. G. , Deeks, J. J. , & Altman, D. G. (2003). Measuring inconsistency in meta‐analyses. BMJ (Clinical Research ed.), 327, 557–560. 10.1136/bmj.327.7414.557 PMC192859 12958120

[cl21190-bib-0028] Higgins, J. P. T. , Thomas, J. , Chandler, J. , Cumpston, M. , Li, T. , Page, M.J. , & Welch, V. A. (Eds.). (2020). *Cochrane handbook for systematic reviews of interventions version 6.1 (updated September 2020)*. Cochrane. www.training.cochrane.org/handbook 10.1002/14651858.ED000142 PMC10284251 31643080

[cl21190-bib-0029] Hirschi, T. (1969). Causes of delinquency. University of California Press

[cl21190-bib-0030] Hoffmann, J. P. , & Dufur, M. J. (2018). Family social capital, family social bonds, and juvenile delinquency. American Behavioral Scientist, 62, 1525–1544. 10.1177/0002764218787020

[cl21190-bib-0031] Hunter, J. E. , & Schmidt, F. L. (2004). Methods of meta‐analysis: correcting error and bias in research findings. Sage.

[cl21190-bib-0032] Jasko, K. , LaFree, G. , & Kruglanski, A. (2016). Quest for significance and violent extremism: The case of domestic radicalization. Political Psychology, 38, 815–831. 10.1111/pops.12376

[cl21190-bib-0033] King, M. , Noor, H. , & Taylor, D. M. (2011). Normative support for terrorism: The attitudes and beliefs of immediate relatives of Jema'ah Islamiyah members. Studies in Conflict & Terrorism, 34(5), 402–417.

[cl21190-bib-0034] Koehler, D. (2013). Family counselling as prevention and intervention tool against “Foreign Fighters”. The German “Hayat” Program. Journal Exit‐Deutschland. Zeitschrift für Deradikalisierung und demokratische Kultur, 3, 182–204.

[cl21190-bib-0035] Koehler, D. (2015). Family counselling, de‐radicalization and counter‐terrorism: the Danish and German programs in context. In S. Zeiger , & A. Aly (Eds.), Countering violent extremism: Developing an evidence‐base for policy and practice (pp. 129–138). Curtin University.

[cl21190-bib-0036] Kraemer, H. C. , Kazdin, A. E. , Offord, D. R. , Kessler, R. C. , Jensen, P. S. , & Kupfer, D. J. (1997). Coming to terms with the terms of risk. Archives of General Psychiatry, 54(4), 337–343. 10.1001/archpsyc.1997.01830160065009 9107150

[cl21190-bib-0037] Kruglanski, A. W. , Gelfand, M. J. , Bélanger, J. J. , Sheveland, A. , Hetiarachchi, M. , & Gunaratna, R. (2014). The psychology of radicalization and deradicalization: How significance quest impacts violent extremism. Political Psychology, 35, 69–93. 10.1111/pops.12163

[cl21190-bib-0038] Lipsey, M. W. , & Wilson, D. B. (2001). Practical meta‐analysis. SAGE publications, Inc.

[cl21190-bib-0039] López‐López, J. A. , Page, M. J. , Lipsey, M. W. , & Higgins, J. P. (2018). Dealing with effect size multiplicity in systematic reviews and meta‐analyses. Research Synthesis Methods, 9, 336–351. 10.1002/jrsm.1310 29971966

[cl21190-bib-0040] Lösel, F. , & Farrington, D. P. (2012). Direct protective and buffering protective factors in the development of youth violence. American Journal of Preventive Medicine, 43(2), S8–S23. 10.1016/j.amepre.2012.04.029 22789961

[cl21190-bib-0041] Lösel, F. , King, S. , Bender, D. , & Jugl, I. (2018). Protective factors against extremism and violent radicalization: A systematic review of research. International Journal of Developmental Science, 12, 89–102. 10.3233/DEV-170241

[cl21190-bib-0042] McCauley, C. , & Moskalenko, S. (2017). Understanding political radicalization: The two‐pyramids model. American Psychologist, 72(3), 205–216. 10.1037/amp0000062 28383974

[cl21190-bib-0043] Moghaddam, F. M. (2005). The staircase to terrorism: A psychological exploration. American Psychologist, 60(2), 161–169. 10.1037/0003-066X.60.2.161 15740448

[cl21190-bib-0044] Murray, J. , Farrington, D. P. , & Eisner, M. P. (2009). Drawing conclusions about causes from systematic reviews of risk factors: The Cambridge Quality Checklists. Journal of Experimental Criminology, 5, 1–23.

[cl21190-bib-0045] Neumann, P. (2013). The trouble with radicalization. International Affairs, 89(4), 873–893. 10.1111/1468-2346.12049

[cl21190-bib-0046] Nivette, A. , Eisner, M. , & Ribeaud, D. (2017). Developmental predictors of violent extremist attitudes: A test of general strain theory. Journal of Research in Crime and Delinquency, 54(6), 755–790. 10.1177/0022427817699035

[cl21190-bib-0047] Pisoiu, D. , & Ahmed, R. (2016). Radicalisation research‐gap analysis. Radicalisation Awareness Network, https://ec.europa.eu/home-affairs/sites/homeaffairs/files/docs/pages/201612_radicalisation_research_gap_analysis_en.pdf

[cl21190-bib-0048] Radicalisation Awareness Network . (2017). Working with families and safeguarding children from radicalization. Step‐by‐step guidance paper for practitioners and policy‐makers. RAN YF&C and RAN H&SC, https://ec.europa.eu/home-affairs/sites/homeaffairs/files/what-we-do/networks/radicalisation_awareness_network/about-ran/ran-h-and-sc/docs/ran_yf-c_h-sc_working_with_families_safeguarding_children_en.pdf

[cl21190-bib-0049] Sageman, M. (2004). Understanding terror networks. University of Pennsylvania Press.

[cl21190-bib-0050] Sampson, R. J. , & Laub, J. H. (1995). Crime in the making: Pathways and turning points through life. Harvard University Press.

[cl21190-bib-0051] Scheff, T. J. (1974). The labelling theory of mental illness. American Sociological Review, 39, 444–452. 4844995

[cl21190-bib-0052] Schils, N. , & Verhage, A. (2017). Understanding how and why young people enter radical or violent extremist groups. International Journal of Conflict and Violence, 11, 1–17. 10.4119/UNIBI/ijcv.473

[cl21190-bib-0053] Schmid, A. P. (2013). Radicalisation, de‐radicalisation, counter‐radicalisation: A conceptual discussion and literature review. The International Centre for Counter‐Terrorism—The Hague, 4(2), 1–91. 10.19165/2013.1.02

[cl21190-bib-0054] Schuurman, B. , & Taylor, M. (2018). Reconsidering radicalization: Fanaticism and the link between ideas and violence. Perspectives on Terrorism, 12(1), 3–22.

[cl21190-bib-0055] Siedler, T. (2006). *Family and politics: Does parental unemployment cause right‐wing extremism?* IZA Discussion Paper No. 2411.

[cl21190-bib-0056] Sikkens, E. , Van San, M. , Sieckelinck, S. , & De Winter, M. (2017). Parental influence on radicalization and de‐radicalization according to the lived experiences of former extremists and their families. Journal for Deradicalization, 12, 192–226.

[cl21190-bib-0057] Sikkens, E. , Sieckelinck, S. , Van San, M. , & De Winter, M. (2017). Parental reaction towards radicalization in young people. Child and Family Social Work, 22, 1044–1053. 10.1111/cfs.12324

[cl21190-bib-0058] Spalek, B. (2016). Radicalisation, de‐radicalisation and counter‐radicalisation in relation to families: Key challenges for research, policy, and practice. Security Journal, 29, 39–52. 10.1057/sj.2015.43

[cl21190-bib-0059] Speckhard, A. , & Akhmedova, K. (2005). Talking to terrorists. Journal of Psychohistory, 33(2), 125–149.

[cl21190-bib-0060] Sterne, J. A. C. , Hernán, M. A. , Reeves, B. C. , Savović, J. , Berkman, N. D. , Viswanathan, M. , Henry, D. , Altman, D. G. , Ansari, M. T. , Boutron, I. , Carpenter, J. R. , Chan, A. W. , Churchill, R. , Deeks, J. J. , Hróbjartsson, A. , Kirkham, J. , Jüni, P. , Loke, Y. K. , Pigott, T. D. , … Higgins, J. P. T. (2016). ROBINS‐I: A tool for assessing risk of bias in non‐randomized studies of interventions. British Medical Journal, 355, i4919. 10.1136/bmj.i4919 27733354 PMC5062054

[cl21190-bib-0061] Sterne, J. A. C. , Savović, J. , Page, M. J. , Elbers, R. G. , Blencowe, N. S. , Boutron, I. , Cates, C. J. , Cheng, H.‐Y. , Corbett, M. S. , Eldridge, S. M. , Hernán, M. A. , Hopewell, S. , Hróbjartsson, A. , Junqueira, D. R. , Jüni, P. , Kirkham, J. J. , Lasserson, T. , Li, T. , McAleenan, A. , … Higgins, J. P. T. (2019). RoB 2: A revised tool for assessing risk of bias in randomised trials. British Medical Journal, 366, l4898. 31462531 10.1136/bmj.l4898

[cl21190-bib-0062] Ventriglio, A. , & Bhugra, D. (2019). Identity, alienation, and violent radicalization. In D. Marazziti , & S. M. Stahl (Eds.), Evil, terrorism and psychiatry (pp. 17–29). Cambridge University Press. 10.1017/9781108569095.005

[cl21190-bib-0063] Wilson, D. B. , Gottfredson, D. C. , & Najaka, S. S. (2001). School based prevention of problem behaviors: A meta‐analysis. Journal of Quantitative Criminology, 17, 247–272.

[cl21190-bib-0069] Williams, J. E. (1984). Secondary victimization: Confronting public attitudes about rape. Victimology, 9, 66–81.

[cl21190-bib-0064] Wolfowicz, M. , Litmanovitz, Y. , Weisburd, D. , & Hasisi, B. (2019). A field‐wide systematic review and meta‐analysis of putative risk and protective factors for radicalization outcomes. Journal of Quantitative Criminology, 10.1007/s10940-019-09439-4

[cl21190-bib-0065] Wolfowicz, M. , Litmanovitz, Y. , Weisburd, D. , & Hasisi, B. (2020). PROTOCOL: Cognitive and behavioral radicalization: A systematic review of the putative risk and protective factors. Campbell Systematic Reviews, 16, e1102. 10.1002/cl2.1102 PMC8356320 37131918

[cl21190-bib-0066] Zych, I. , Farrington, D. P. , Ribeaud, D. , & Eisner, M. P. (2021). Childhood risk factors for adolescent offending: A cross‐national comparison based on official records in London, Pittsburgh and Zurich. Journal of Developmental and Life‐Course Criminology, 91, 527–545. 10.1007/s40865-021-00167-7

[cl21190-bib-0067] Zych, I. , Gómez‐Ortiz, O. , Fernández Touceda, L. , Nasaescu, E. , & Llorent, V. J. (2020). Parental moral disengagement induction as a predictor of bullying and cyberbullying: Mediation by children's moral disengagement, moral emotions, and validation of a questionnaire. Child Indicators Research, 13, 1065–1083. 10.1007/s12187-019-09670-2

